# Analysis of Fc-dependent effector functions of anti-malaria circumsporozoite protein antibodies

**DOI:** 10.1128/spectrum.00863-25

**Published:** 2025-07-23

**Authors:** Erin Stefanutti, Rashmi Ramani, Bradley Whitener, Ha Dang, Simon Bélanger, Logeshwaran Somasundaram, Karen Cortina, Anna De Marco, Tommy Tam, Qingqing Chai, Elisabetta Cameroni, Rajesh Gupta, Michael A. Schmid, Jessica L. Miller, Anna Brotcke Zumsteg, Lisa A. Purcell, Lisa L. Drewry

**Affiliations:** 1 Vir Biotechnology 630422 , San Francisco, California, USA; 2 Vir Biotechnology, Bellinzona, Switzerland; Barcelona Centre for International Health Research (CRESIB, Hospital ClÃ­nic-Universitat de Barcelona), Barcelona, Spain

**Keywords:** malaria, antibody functions, monoclonal antibodies, effector functions, antimalarial agents

## Abstract

Antibodies targeting the malaria circumsporozoite protein (CSP) can prophylactically protect against malaria by targeting *Plasmodium* parasites before they establish symptomatic blood-stage disease. Engineering the antibody Fc region to more effectively engage immune effector functions has produced therapeutic antibodies with enhanced potency against viral and oncological targets. However, whether Fc-dependent immune effector functions can contribute to the protection of malaria CSP mAbs or be further enhanced via engineering has been limitedly tested. Here, we report that Fc-dependent effector functions are required for achieving maximal protection via prophylactic treatment with the CSP mAb 317. We further report that Fc engineering modulated the activity of multiple CSP mAbs in multiple *in vitro* assays of effector function. Our studies revealed that the mAbs L9 and CIS43 were more potent drivers of antibody-dependent phagocytosis, NK activation and killing, and complement deposition. In contrast, 317, but not L9 and CIS43, drove enhanced activation of CSP-responsive T-cells after DC acquisition of mAb-complexed antigens. Collectively, our data suggest that effector function represents an important mechanism of anti-CSP antibodies with the potential to enhance activity through Fc engineering.

**IMPORTANCE:**

Malaria disease imposes a major burden on global health, causing over half a million annual deaths. Recent clinical trials in humans have shown that therapeutic antibodies can provide prophylactic protection against malaria in target populations. However, the cost of goods for therapeutic antibodies is high, and the malaria disease burden is concentrated in resource-challenged regions. Engineering the antibody Fc domain to more efficiently engage the immune system is an appealing strategy to increase the potency of therapeutic antibodies but has been minimally tested for malaria. Here, we present evidence that the Fc domain of malaria therapeutic antibodies can confer protection in animal models and can be engineered for more potent stimulation of diverse parasite-targeting immune responses.

## INTRODUCTION

Malaria is a scourge on global health, causing over 200 million cases and 600,000 deaths annually. Recent approval of the R21/Matrix-M vaccine after Ph3 trial (1) and reports of prophylactic protection by the therapeutic antibodies L9 and CIS43 in human trials (2, 3) represent exciting progress in the fight against malaria.

Technological advancements allowed therapeutic antibodies to play a crucial role in early control of the COVID-19 pandemic and may also enable antibodies to play a meaningful role in controlling malaria disease burden (4). Encouragingly, monoclonal antibodies against the malaria circumsporozoite protein (CSP) target have shown promising ability to protect against malaria challenge, in both human (2, 3, 5) and animal studies (6–9).

Antibodies protect against infection via mechanisms dependent on two distinct domains: i) binding of the antibody Fab domain to target antigen can directly neutralize pathogens and ii) binding of the antibody Fc domain to leukocyte-expressed Fc-γ receptors (FcgRs) can stimulate a multitude of immune effector mechanisms with anti-pathogenic activity (10). Correlates of protection for the CSP-targeting malaria vaccines and therapeutic antibodies remain poorly understood. Systems serology analyses identified effector function as a protective correlate of the CSP-targeting RTSS/AS01 vaccine (11, 12). However, limited studies have tested whether CSP mAbs protect solely via Fab-mediated neutralization or also achieve protection through Fc-dependent immune effector functions.

In contrast, evidence that antibody Fc-dependent immune effector functions contribute to therapeutic antibody efficacy has been shown against pathogens including SARS-CoV-2 (13–15), HIV (16), influenza (17, 18), and *Mycobacterium tuberculosis* (19). Advancements in the mechanistic understanding of Fc-dependent effector functions have also enabled the rational design of Fc-enhanced therapeutic antibodies against oncological targets with clinical success (20). Leveraging Fc-dependent immune effector functions to optimize anti-malarial antibody therapeutics offers a potential avenue to increase the potency, extend half-life, and reduce cost of goods and thereby enhance the tractability of this technology in resource-limited areas where the disease burden is concentrated (21).

Here, we report a comprehensive investigation into Fc-dependent immune effector functions of therapeutic CSP antibodies. Our studies used a humanized mouse model to reveal a role for Fc-dependent immune effector functions in generating maximal protection conferred in the previously described CSP major repeat targeting antibody, 317 (22). Intriguingly, Fc dependence of protection was not observed for the most clinically advanced CSP antibody, L9. We followed-up on these results with a systematic characterization of the ability of CSP mAbs to induce immune effector functions *in vitro*. To probe whether Fc-dependent effector functions vary across CSP mAbs, we incorporated an additional CSP mAb, CIS43 into our *in vitro* characterization. We report the varied baseline ability of L9, CIS43, and 317 to promote immune effector functions. Excitingly, we found that engineering of the Fc domain of all tested CSP mAbs can functionally modify their effector function potency. In total, our findings suggest that Fc-engineering CSP mAbs has the potential to increase their potency and tractability as prophylactic therapeutics in malaria-endemic areas.

## RESULTS

### Fc domain of the NANP repeat-binding antibody 317 was crucial for achieving maximal *in vivo* protection

We first asked whether the Fc domain of CSP-binding antibodies contributes to protection. To this end, we challenged humanized FcgR mice with transgenic *Plasmodium berghei* parasites engineered for replacement of the native *P. berghei* CSP with the full-length CSP of *Plasmodium falciparum* (*Pf*CSP) and dual reporters for luciferase and GFP (Pb-*Pf*CSP-*Luc-GFP*) (23). This pairing enabled testing of clinically relevant human antibodies that bind *Pf*CSP in a host bearing species-matched FcgRs. All CSP mAbs tested for protection in our studies harbored the half-life extending M428L/N434S (LS) Fc mutation, as do all CSP antibody candidates tested in humans (2, 3, 24).

**Fig 1:**
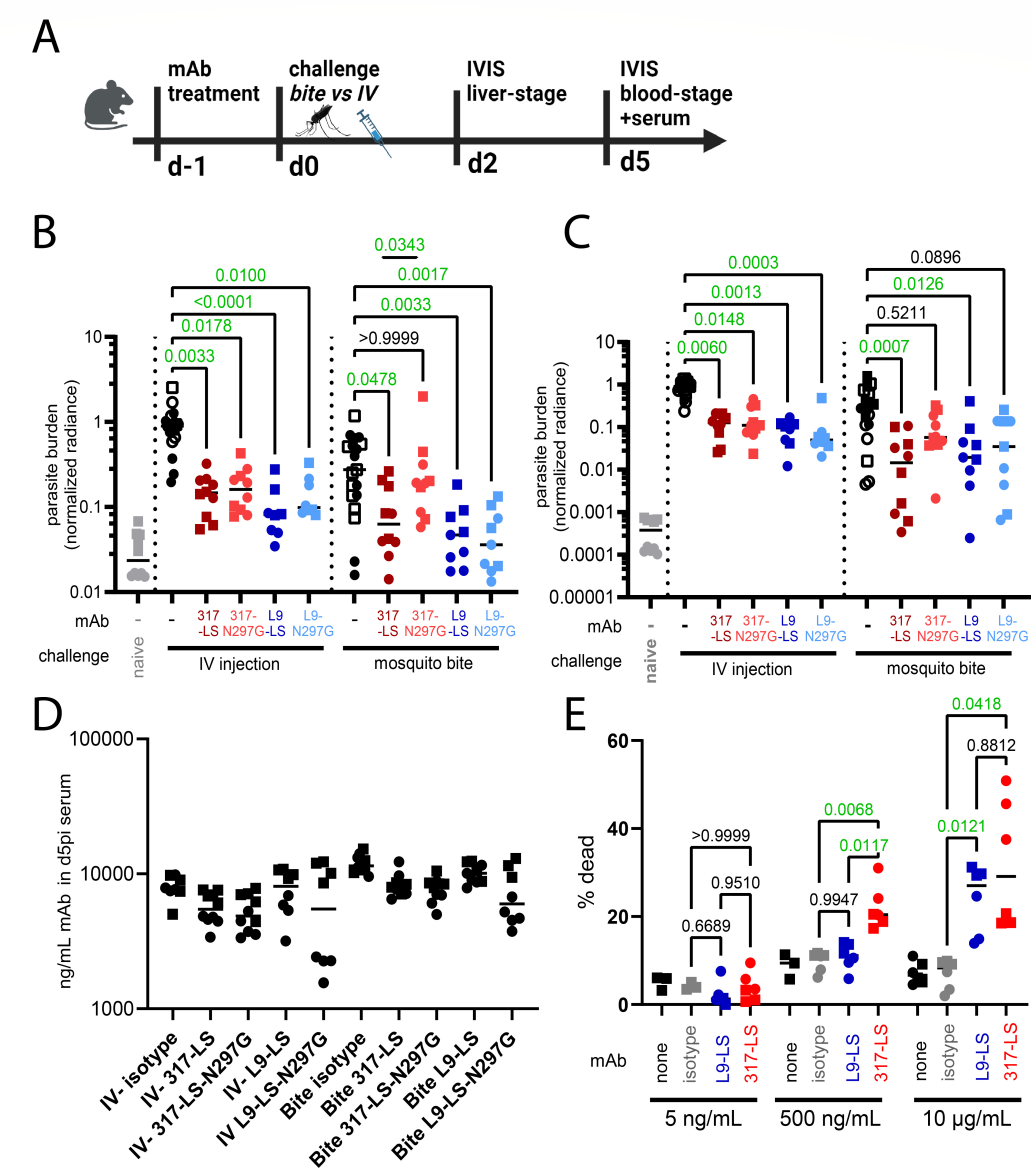
Fc domain of CSP-binding mAb 317, but not L9, was required for maximal protection. (**A**) Schematic of the challenge model. (**B–C**) Parasite burden was measured by IVIS optical imaging at d2pi (**B**) and d5pi (**C**). Control mice were treated with either PBS (closed markers) or an LS-modified IgG1 isotype control antibody (open markers). Radiance was measured in an ROI enclosing the liver (**B**) or whole mouse (**C**) to quantify parasite burden. (**D**) Concentration of CSP mAbs in serum collected at d5pi. (**E**) Percentage of purified salivary gland sporozoites staining as nonviable after co-incubation with indicated mAbs. (**B–E**) Pooled data from two independent experiments are shown, with values normalized to the value of the mAb-untreated IV challenge control group from each experiment. Matched marker shapes (circles and squares) indicate data originating from the same experiment. Results of one-way ANOVA with Kruskal-Wallis post-test are indicated for B, C, and one-way ANOVA with Brown-Forsythe and Welch post-test are indicated for E; all calculated *P*-values are shown, with statistically significant comparisons highlighted with green text.

Mice were challenged with Pb-*Pf*CSP-*Luc-GFP* parasites 1 day after antibody treatment, and parasite burden was assessed via IVIS optical imaging at late liver-stage (2 days post-infection; d2pi) (Fig. 1A). As expected, treatment with 317-LS and L9-LS mAbs protected against challenge by either IV parasite inoculation or bite of infected mosquitoes (Fig. 1B). In the IV inoculation model, introduction of an N297G mutation shown to ablate binding to all FcgRs (25) did not eliminate the protection of 317-LS or L9-LS (Fig. 1B). In contrast, in the mosquito bite model, FcgR-binding-ablated 317-LS-N297G did not yield protective effects, while L9-LS-N297G maintained protection (Fig. 1B).

We also quantified parasite burden during early blood stage (d5pi) to test whether differential liver-stage burden continued into blood stage. LS and LS-N297G variants of both L9 and 317 reduced the parasite burden at d5pi after IV challenge, and 317-LS, but not 317-LS-N297G, reduced the burden at d5pi after mosquito bite challenge (Fig. 1C). L9-LS reduced burden at d5pi after mosquito bite, while L9-LS-N297G-treated mice showed a trend to reduced burden that was not statistically significant (*P* = 0.0896) (Fig. 1C).

To test if the differences observed in protection could be explained by unfavorable pharmacokinetics or inaccurate dosing, we quantified the antibody serum concentration in d5pi serum. The human monoclonal antibodies 317-LS and 317-LS-N297G were present in serum at similar levels, suggesting the loss of protection in the 317-LS-N297G variant cannot be explained by improper dosing or a more rapid clearance *in vivo* (Fig. 1D). Curiously, in a single experiment, L9-LS-N297G protected well from IV challenge despite being present at reduced levels (Fig. 1D), underscoring the competence of L9-LS to provide protection in the absence of robust FcgR binding.

In total, these results suggest that Fc-mediated effector functions are critical to the ability of 317 mAb to confer protection against malaria upon mosquito bite challenge. Intriguingly, this was not universal for all CSP-targeting mAbs as our experiments did not discern reduced protection by L9-LS mAb upon introduction of the Fc silencing N297G modification.

One potential explanation for the discordance between L9-LS and 317-LS is that L9-LS could neutralize parasites so potently that little room remains for Fc-domain-dependent activities to further improve protection. A recent report identified lethal precipitation of CSP from the parasite surface as a correlate of *in vivo* protection by CSP mAbs (26). Hence, we compared parasite neutralization via direct killing by L9-LS and 317-LS. Both mAbs exhibited dose-dependent cytotoxicity, with robust killing at 10 microgram/mL mAb (Fig. 1E). However, in our assay, 317-LS was a more potent killer of parasites, with 317-LS but not L9-LS exhibiting cytotoxicity at a 500 ng/mL concentration (Fig. 1E). This finding is inconsistent with that of a model where the superior neutralizing ability of L9-LS eclipses any contribution of Fc-dependent functions to L9-LS-conferred protection as L9-LS was no more potent at neutralizing parasites than 317-LS.

These observations prompted us to undertake a mechanistic analysis of the potential contributions of antibody Fc domain-driven immune effector function activities to protection conferred by CSP antibodies. To this end, we undertook a comprehensive *in vitro* evaluation of the ability of three distinct CSP-binding mAbs to induce immune effector functions, with each mAb tested with a series of Fc domains harboring modifications designed to imprint a unique FcgR binding profile.

### Modifying the Fc domain of CSP mAbs generated a panel of mAbs with engineered FcgR-binding profiles

The role of antibody Fc-driven immune effector function in conferring protection against malaria remains largely unexplored. Hence, we designed a panel of Fc modifications with the aim of targeting binding to FcgRs implicated in diverse immune effector functions.

Our Fc modification panel included the following: an N297G mutation that abrogates binding to all Fc receptors and C1q (25, 27); a G236A/A330L/I332E (GAALIE) mutation, known to enhance binding to FcgR2a, FcgR3a, and FcgR3b and decrease C1q binding (18, 28); a G236A mutation that enhances binding to FcgR2a while decreasing binding to FcgR3a/b (29); an S267E/H268F/S324T (EFT) modification that enhances binding to complement C1q protein (30); and afucosylated (AFUC) antibodies expected to strongly bind FcgR3a/b (31–33). All clinically tested CSP mAbs include the half-life, extending the LS mutation (2, 3, 24, 34). Hence, our Fc modifications were made in addition to the LS mutation. Fc-engineered variants of the CSP mAbs L9-LS and CIS43-LS were tested as representatives of mAbs preferentially binding the N-terminal-NANP repeat junction and junction-adjacent NVDP repeats of CSP (9, 35, 36), while 317-LS was tested for its distinct preferential binding in the NANP major repeats of CSP (22).

**Fig 2:**
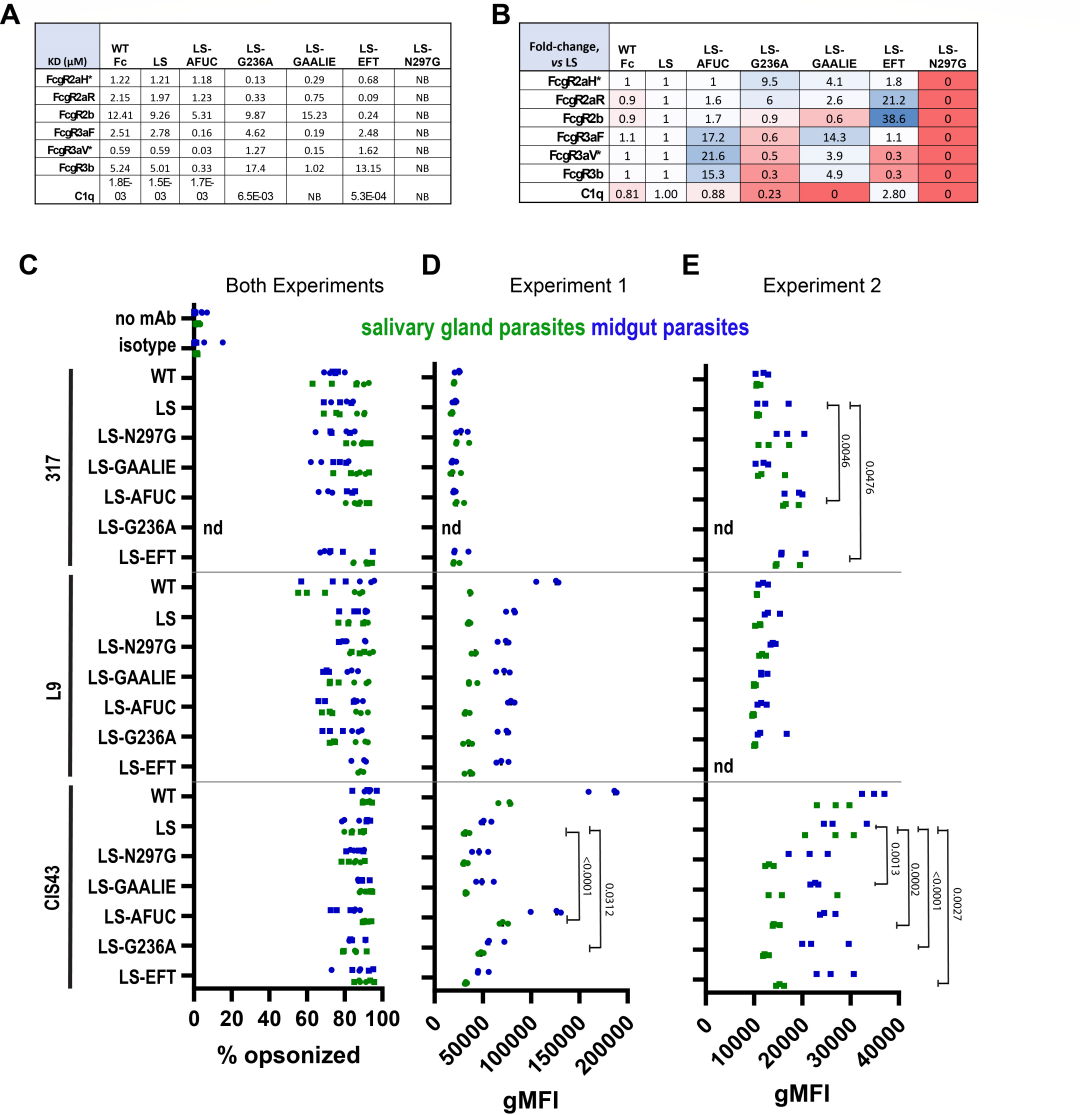
Binding analysis of Fc-engineered CSP mAbs. (**A–B**) Binding affinity of Fc-engineered CIS43 variants to a panel of FcgRs was measured by SPR and to C1q by Octet BLI. (**A**) shows binding affinity, and (**B**) shows fold-change in affinity, relative to CIS43-LS mAb, with red shading highlighting increased affinity and blue decreased affinity. Data shown are average values calculated from two technical replicates, except for two receptors (*), FcgR2aH and FcgR3aV, calculated from a single technical replicate. KD values could not be quantitated for the N297G variant due to undetectable binding to all receptors. (**C–E**) Binding of mAbs to live parasites upon co-incubation with 5 ng/mL mAb was assessed by flow cytometry. (**C**) Percent of parasites harvested from either mosquito salivary glands (green markers) or midguts (blue markers) opsonized by indicated mAbs is shown with pooling data from two independent experiments, each with three technical replicates. (**D, E**) gMFI corresponding to mAb binding intensity is shown for opsonized parasites in two independent experiments. All statistically significant (*P* < 0.05) comparisons were assessed (**C–E**) using a two-way ANOVA using Sidak’s multiple comparison test to assess differences between Fc variants compared to the LS control Fc, regardless of whether parasites originated from the midgut or salivary glands. Fc mAb variants not tested due to limited parasite numbers, and are indicated by nd (not done).

We first asked whether Fc-engineered variants of the mAb CIS43 exhibited the predicted changes in FcgR binding affinities. As expected, we observed that the N297G modification abrogated binding to all tested receptors (Fig. 2A and B). The EFT mutation enhanced binding to C1q and also FcgR2a and FcgR2b (Fig. 2A and B). Afucosylated CIS43 exhibited enhanced binding to FcgR3a and FcgR3b and modestly enhanced binding to FcgR2a and FcgR2b (Fig. 2A and B). CIS43-LS-G236A, as expected, showed enhanced affinity to FcgR2a (Fig. 2A and B). CIS43-GAALIE bound FcgR2a, FcgR3a, and FcgR3b with enhanced affinity and exhibited the expected decreased C1q affinity (Fig. 2A and B). Comparison of CIS43 wild-type and CIS43-LS showed only modest differences in affinity (Fig. 2A and B). We posit that Fc-modified variants of the CSP mAbs, 317-LS and L9-LS, likely exhibit similar FcgR-binding profiles, but did not experimentally test this prediction.

Our working model of Fc engineering posits that manipulations to the antibody Fc domain should modulate FcgR binding without altering antibody Fab binding to the antigenic target. We tested this prediction by using flow cytometry to measure the binding of our Fc-engineered CSP mAbs to live parasites. To test if previously reported differences in the folding and proteolytic processing of CSP (37) influence mAb binding, we assessed binding to fully mature and infectious parasites harvested from the mosquito salivary glands and immature parasites harvested from the mosquito midgut. Evaluating parasites as either opsonized/not-opsonized, we observed consistently high opsonization by all mAbs of both salivary gland and midgut parasites, with no apparent compromise in opsonization by any Fc-engineered mAb variants (Fig. 2C).

To probe for subtle opsonization differences, we used the gMFI of mAb-detecting secondary antibodies as a proxy for the mAb binding density. Across independent experiments, gMFI absolute values varied substantially (Fig. 2D and E). We did not observe any marked trends toward decreased binding of Fc-engineered CSP mAbs, compared to their respective 317-LS, L9-LS, and CIS43-LS controls (Fig. 2C through E). However, for the mAbs L9 and CIS43, there was a trend toward denser opsonization of midgut parasites, compared to mature salivary gland parasites, which was not observed for the mAb 317 (Fig. 2D and E). Notably, we also observed more dense binding of both midgut and salivary gland parasites by CIS43 wild-type mAb, compared to all variants with an LS-bearing Fc (Fig. 2D and E). Despite some differences that were observed for CIS43 in two individual experiments (Fig. 2D and E) between the LS-Fc and its further engineered versions, no significant differences in parasite binding were observed overall (Fig. 2C).

These studies confirmed that Fc modifications altered the binding of CSP mAbs to Fc receptors without any major perturbation in binding to CSP expressed on live parasites. We next tested if modulating FcgR binding via antibody Fc engineering produced functional consequences in antibody-driven immune effector functions. To enable a high-throughput strategy, we leveraged beads and plates coated with recombinant CSP protein (rCSP) for these assays.

### Differential enhancement of antibody-dependent opsonic phagocytosis by CSP mAbs

**Fig 3:**
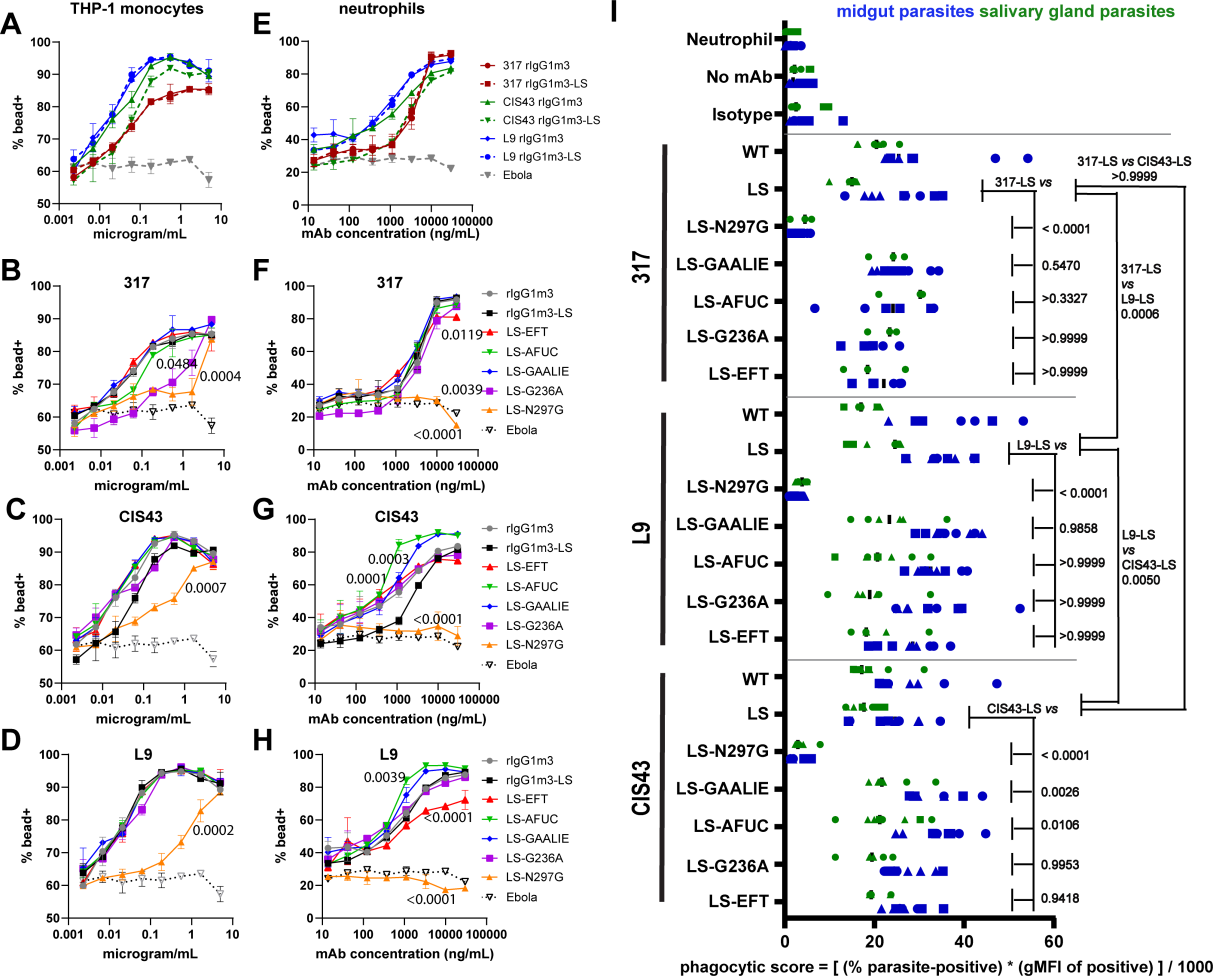
Antibody-dependent phagocytosis was influenced by the Fc region of CSP mAbs. (**A–H**) Phagocytosis of rCSP-coated beads by THP-1 monocytes (**A–D**) or primary human neutrophils (**E–H**) was measured via flow cytometry, following co-incubation of mAbs at indicated dilution series with rCSP-coated beads. Data show average values from two technical replicates, with error bars indicating SD. Two separate donors were used to source neutrophil technical replicates. *P* values in B–D and F–H indicate statistically significant differences between the area under the curve of the respective curve of the Fc variant compared to the LS control Fc, using one-way ANOVA with Dunnett’s multiple comparisons test (**I**) Phagocytosis of live Pb-*Pf*CSP-*Luc-GFP* parasites by primary human neutrophils, following co-incubation with 5 ng/mL mAbs. Plots show data from 2 to 3 independent experiments for each mAb, each with three technical replicates. Matching marker shapes (circles, squares, and triangles) indicate data originating from the same experiment. *P*-values in black indicate calculated results of Sidak’s multiple comparisons tests following a two-way ANOVA comparing differences between mAb-LS Fc variants to the LS control, regardless of whether parasites originated from the midgut or salivary gland.

A flow cytometry-based assay was used to quantify mAb promotion of opsonic phagocytosis of beads coated with rCSP protein. We first used THP-1 monocytes as a model phagocyte. Relative to a control mAb against an irrelevant Ebola protein target, we found that the mAbs 317, L9, and CIS43 all drove the enhanced uptake of rCSP-coated beads, with the introduction of the half-life LS modification not impacting 317 or L9 activity, while slightly compromising CIS43 functionality (Fig. 3A). Notably, the junctional targeting mAbs L9 and CIS43 more potently enhanced THP-1 phagocytosis than NANP-repeat-binding 317 mAb (Fig. 3A). Abrogation of FcgR binding via introduction of an N297G Fc mutation reduced phagocytic enhancement by all CSP mAbs (Fig. 3B through D). We did not observe increased enhancement of THP-1 phagocytosis in any of our Fc-engineered CSP mAbs (Fig. 3B through D), in contrast to our literature-based prediction that Fc modifications that enhance FcgR2a binding would show increased functionality (29, 38).

Prior work has highlighted neutrophils as the predominant mediator of sporozoite clearance from peripheral blood (39). Hence, we next tested primary peripheral blood neutrophils as a more physiologically relevant phagocyte. We observed that all CSP mAbs induced neutrophil-mediated phagocytosis of rCSP-coated beads compared to an irrelevant Ebola mAb control (Fig. 3E). However, L9 and CIS43 mAbs were more potent at driving neutrophil phagocytosis than the 317 mAb (Fig. 3E). Activity of all mAbs was disrupted by the FcgR-binding-ablating N297G Fc mutation (Fig. 3F through H). For L9 and CIS43 mAbs, variants featuring a GAALIE Fc mutation or afucosylated (AFUC) Fc were most potent at driving neutrophil phagocytosis (Fig. 3G and H). Curiously, for the 317 mAb, GAALIE and afucosylated variants did not enhance neutrophil phagocytosis (Fig. 3F).

To probe the translatability of these findings to a system with greater physiological relevance, we developed a flow cytometry-based assay that measured uptake of mAb-opsonized live parasites by primary human neutrophils. The live parasite phagocytosis assay revealed that 317, L9, and CIS43 mAbs all promoted neutrophil uptake of parasites (Fig. 3I). As expected, ablation of FcgR binding with an N297G mutation abrogated mAb-driven phagocytosis for all three CSP mAbs (Fig. 3I). For the mAb CIS43-LS, we detected significant further enhancement of phagocytosis by the introduction of GAALIE or AFUC modifications (Fig. 3I), consistent with the performance of CIS43-LS-GAALIE and CIS43-LS-AFUC in our rCSP-coated bead phagocytosis assay (Fig. 3G). Enhanced performance in the live parasite phagocytosis assay was not detected for Fc-engineered variants of L9-LS and 317-LS (Fig. 3I).

L9-LS drove significantly more parasite phagocytosis than 317-LS and CIS43-LS mAbs (Fig. 3I), again consistent with the performance of L9-LS as the most potent mAb in the rCSP-coated bead assay (Fig. 3A and E). In contrast to the rCSP-coated bead phagocytosis assay, our live parasite assay did not discern a significant difference between the potency of CIS43-LS and 317-LS in driving phagocytosis (Fig. 3I). The potency of all tested CSP mAbs in enhancing phagocytosis was superior against live parasites than rCSP beads. Specifically, in the live parasite assay, 5 ng/mL mAb drove robust antibody-dependent phagocytosis of live parasites (Fig. 3I). In contrast, in the rCSP-coated bead assay, dose-dependent mAb-induced phagocytic activity was clearly detected at concentrations above 1 µg/mL, but overwhelmingly lost at concentrations under 100 ng/mL (Fig. 3E).

We also used our live parasite phagocytosis assay to compare the uptake of fully mature and infectious salivary gland parasites to immature midgut parasites. We observed a consistent trend toward more uptake of immature midgut parasites, vs mature salivary gland parasites, across all three CSP mAbs (Fig. 3I). This finding supports the functional relevance of the more efficient opsonization of immature midgut parasites we observed with our live parasite binding assay (Fig. 2C through E).

In total, we noted strong concordance between the findings of our assays measuring phagocytosis of rCSP-coated beads and live parasites. In both assays, L9-LS was the most potent mAb, and functional enhancement was detected upon introduction of GAALIE or AFUC Fc modifications to CIS43-LS (Fig. 3). The rCSP-coated bead phagocytosis assay revealed a greater number of subtle differences between Fc-engineered CSP mAbs, likely due to the tractability of testing a wider range of mAb concentrations with this approach. To maximize our ability to detect functional consequences of Fc engineering, we focused our efforts going forward on rCSP approaches amenable to higher-throughput experiments.

### Differential enhancement of antibody-dependent NK cell activation by CSP mAbs

**Fig 4:**
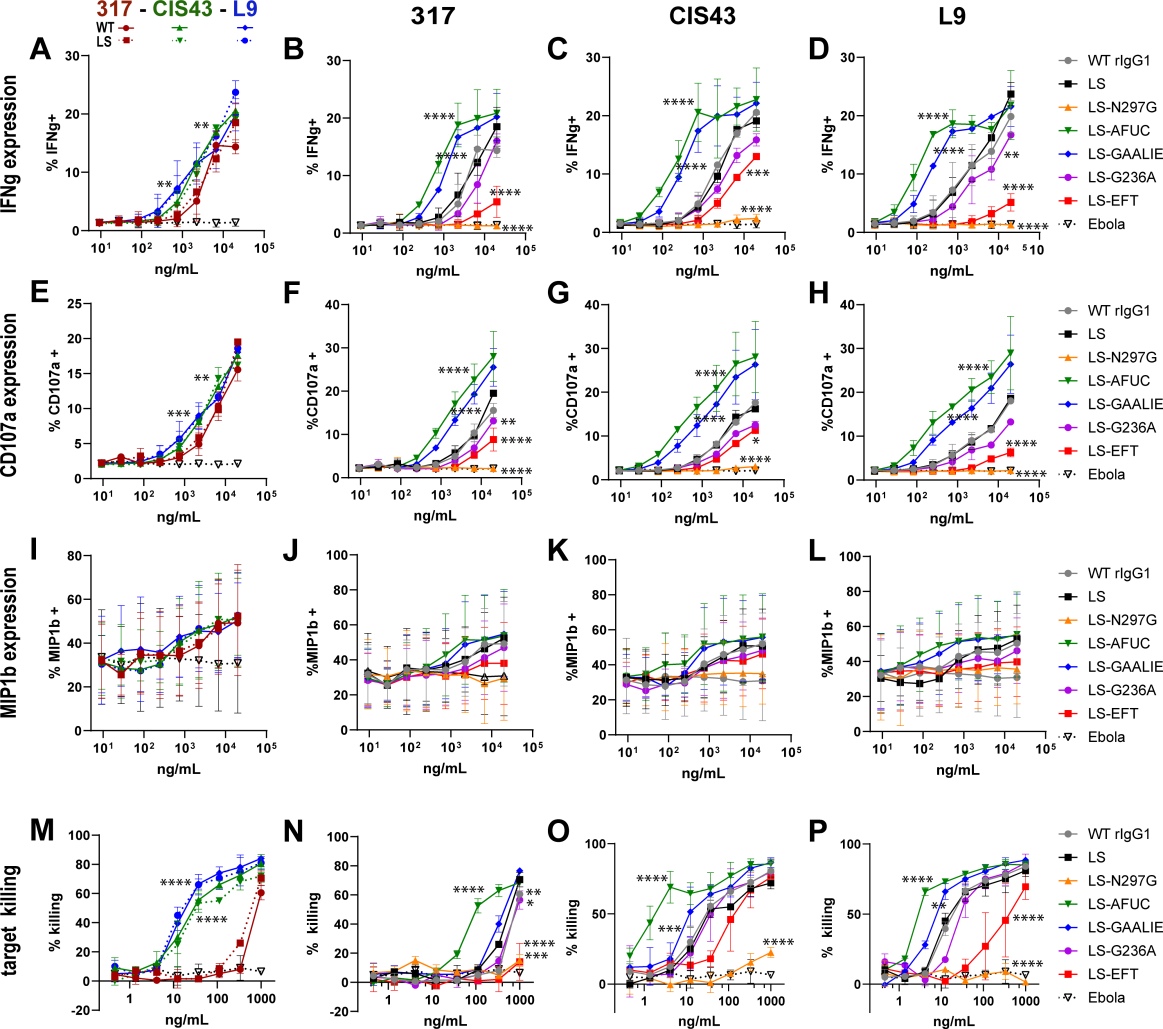
Antibody-dependent NK cell activation and killing were influenced by the Fc region of CSP mAbs. (**A–L**) CSP antigen-induced expression of NK cell activation markers IFN-γ (**A–D**), CD107a (**E–H**), and MIP1-β (**I-L**) was assessed in NK cells following 5 h of co-incubation of NK cells on rCSP-coated plates pre-incubated with indicated mAbs. (**M–P**) Specific killing of rCSP-pulsed target cells by NK cells after 4 h co-incubation. All data shows average values of two technical replicates, and error bars indicate SD. NK cells were sourced from unique leukopak donors for each technical replicate. Statistical comparisons of CIS43 or L9 vs 317 with wild-type Fc used two-way ANOVA with Sidak’s (A, E, I, and M), and of LS-Fc variants vs the LS-Fc of each mAb used Dunnett’s correction (B–D, F–H, J–L, and N–P) for multiple comparisons. Level of significance: *P < 0.05; **P < 0.01; ***P < 0.001; ****P < 0.0001.

We quantified NK cell activation after co-incubation of rCSP-coated plates with Fc-engineered CSP mAbs with a flow cytometry assay using expressions of IFN-γ, CD107a, and MIP1-β as activation reporters. In both wild-type and LS-modified formats, the mAbs 317, CIS43, and L9 all drove NK activation, as evidenced by the enhanced expression of all measured activated markers, compared to an irrelevant mAb control (Fig. 4A, E and I). IFN-γ and CD107a expressions showed robust dynamic ranges of mAb-driven NK activation (Fig. 4A and E); MIP1-β expression was less clear due to the high variability between technical replicates (Fig. 4I). Comparing Fc-engineered variants to 317-LS, CIS43-LS, and L9-LS controls, we observed enhanced NK activation in samples where mAbs harbored the FcgR3a-increasing modifications AFUC and GAALIE (Fig. 4B through D and F through H). Introduction of G236A and EFT modifications that decreased FcgR3a binding (Fig. 2A) led to decreased activation of NK cells (Fig. 4B through D and F through H). We finally measured NK cell functionality by comparing antigen-specific target cell killing by CSP mAbs. In this killing assay, we observed superior potency of the mAbs L9 and CIS43 over 317, regardless of the LS modification (Fig. 4M). This contrasted the observation of the NK activation assay, in which we observed only a subtle trend toward less activation via 317 (Fig. 4A and E). We also observed more potent killing by mAbs harboring AFUC and GAALIE modifications and less potent killing by mAbs harboring G236A and EFT modifications compared to the mAbs harboring the LS-Fc (Fig. 4N through P).

### Differential enhancement of antibody-dependent complement deposition by CSP mAbs

**Fig 5:**
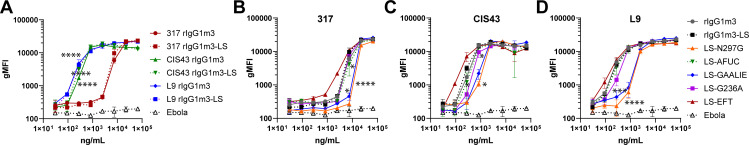
Antibody-dependent complement deposition was influenced by the Fc region of CSP mAbs. (**A–D**) Deposition of C3 complement protein on rCSP-coated beads was detected by flow cytometry, following 50 min incubation with indicated mAbs. Data plots show gMFI for C3 detection antibody from two technical replicates; error bars indicate SD. Statistical comparisons of CIS43 vs L9 vs 317 with wild-type Fc used two-way ANOVA with Sidak’s (**A**) and of LS-Fc variants vs the LS-Fc of each mAb used Dunnett’s correction (B, C, and D) for multiple comparisons. Level of significance: *P < 0.05; **P < 0.01; ***P < 0.001; ****P < 0.0001.

A flow cytometry-based assay was used to quantify the deposition of C3 complement protein on rCSP-coated beads pre-incubated with CSP mAbs. The complement deposition assay revealed enhancement of C3 deposition by all CSP mAbs, relative to control (Fig. 5A). The L9 and CIS43 mAbs enhanced complement deposition with higher potency than the 317 mAb, regardless of the introduction of the LS modification (Fig. 5A). Activity of all CSP mAbs in driving complement deposition was diminished by the introduction of the FcgR-binding-ablating modification N297G (Fig. 5B through D). Introduction of the C1q-binding enhancing Fc modification EFT (Fig. 2A and B) enhanced complement deposition by all CSP mAbs (Fig. 5B through D). We also detected reduced complement deposition in the G236A and GAALIE Fc modifications (Fig. 5B through D), which reduced or respectively abrogated binding to C1q, as observed in our BLI binding analysis (Fig. 2A and B).

### Functionality of CSP mAbs in promoting T-cell activation

**Fig 6:**
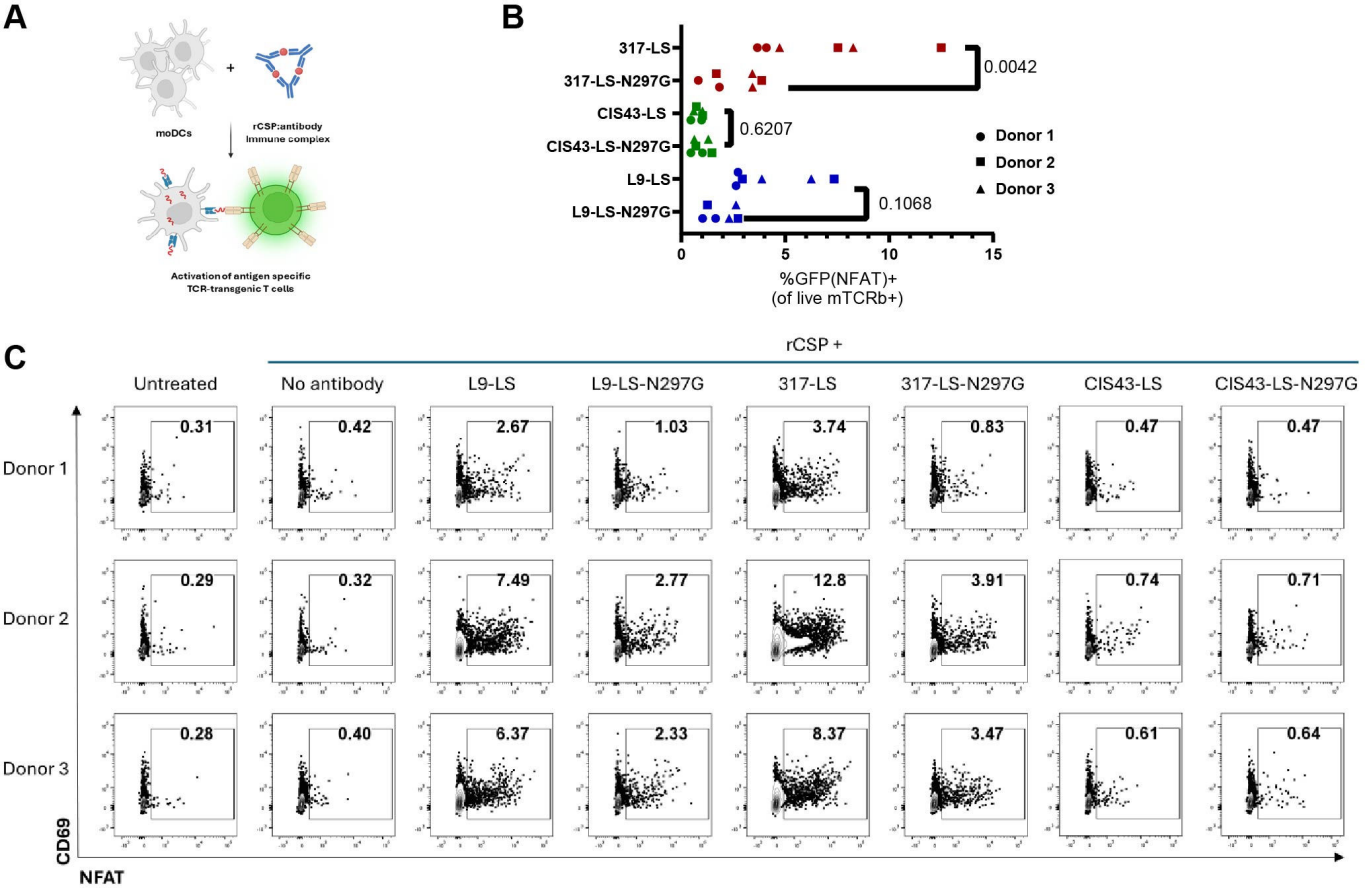
Activation of CSP-specific reporter Jurkat line by CSP antibody immune complexes. (**A–C**) A CSP-responsive reporter Jurkat T-cell line was used to assess the ability of immune complexes formed of rCSP antigen and CSP mAbs to stimulate T-cell activation. Assay set-up is schematized in (**A**), and representative flow plots shown in (**C**). (**B**) Percent-activated CSP reporter T-cells in two independent experiments. Each experiment included three biological replicates of PBMCs sourced from unique donors (donors indicated by marker shapes). *P*-values indicate the results of Sidak’s multiple comparison test with two-way ANOVA, comparing LS and LS-N297G variants of each mAb.

We finally asked if any of the CSP mAbs in our study could induce enhanced T-cell activation, as reported in a recent study with influenza mAbs (18). To this end, we cloned a previously described CSP-responsive TCR (40) into a fluorescent Jurkat CD4 T-cell NFAT-GFP reporter system (41). We then developed an assay where rCSP antigen and CSP mAbs immune complexes are co-incubated with moDC for presentation, and then a final incubation with CSP-responsive Jurkat cells reveals activation via fluorescence of the NFAT-GFP reporter (Fig. 6A). This assay revealed differences in CSP mAb functionality at enhancing T-cell activation upon complexing with the rCSP antigen. 317-LS was the only tested mAb to induce activation of the CSP reporter Jurkat with significant activity over the FcgR-binding-ablated 317-LS-N297G variant (Fig. 6B and C). L9-LS showed a non-statistically significant trend toward activation of CSP TCR reporter Jurkats, compared to the L9-LS-N297G control, while no differences were detected in the activity of CIS43-LS and CIS43-LS-N297G in this assay (Fig. 6B and C). Curiously, despite generating moDC for this assay with donors all matching the mapped haplotype of this TCR (40), we observed substantial between-donor variability in this assay (Fig. 6B and C).

## DISCUSSION

Here, we report an investigation of the potential for enhancing anti-malarial protection by CSP antibodies via augmentation of Fc-dependent immune effector function activity. Using a dual-transgenic parasite-mouse model to test the efficacy of antibodies targeting PfCSP, we first revealed that Fc-dependent immune effector functions were required for maximal protection by the CSP mAb, 317-LS, but not the more clinically advanced L9-LS mAb (Fig. 1A through C). A comprehensive *in vitro* characterization of the Fc effector function capacity of a panel of CSP mAbs harboring modifications in the Fc domain that modulate binding to FcgRs indicated that the CSP mAbs L9-LS, CIS43-LS, and 317-LS were all capable of enhancing innate immune activity directed at CSP antigens. These assays included antibody-dependent phagocytosis by monocytes and neutrophils (Fig. 3), NK cell activation and killing (Fig. 4), and complement deposition (Fig. 5). In the phagocytosis, NK cell-mediated killing, and complement deposition assays, the mAbs L9-LS and CIS43-LS more potently drove enhanced functionality than the 317-LS mAb (Fig. 3 to 5). In the only assay measuring potential bridging to adaptive immunity, we observed enhanced T-cell activation when DC antigen presentation occurred in the presence of immune complexes formed by 317-LS mAb and CSP antigen, but not L9-LS or CIS43-LS mAbs (Fig. 6). We observed that engineering the Fc domain of all tested CSP mAbs modulated binding to FcgRs (Fig. 2) and enhanced functionality in multiple innate immune effector mechanisms *in vitro* (Fig. 3 to 5).

We tested *in vivo* whether an intact CSP mAb Fc domain is required for effective protection by CSP mAbs. Our work shows a requirement for intact Fc-dependent effector function for the mAb 317-LS to achieve maximal prophylactic protection in preclinical models. We cannot exclude any role for the Fc domain in L9-LS-mediated protection due to the limited number of doses tested in our study. However, our work strongly supports a model where Fc-dependent effector function more strongly drives the protection of 317-LS compared to L9-LS. A very recent and complementary publication by Kisalu et al. did not observe significant differences for protection by the CIS43-LS anti-CSP mAb on malaria parasite burden in a mouse model, regardless of whether the Fc was intact or carried the LALA mutation that abrogates FcgR binding (42). The authors observed, however, that engineering the Fc of CIS43-LS to increase FcgR3a and 3b binding had the potential to reduce the liver-stage parasite burden in this model (42). In contrast to CIS43, the authors found that engineering the Fc to enhance or abrogate FcgR binding did not alter the protective capacity of the L9-LS mAb (42), which is in line with our data presented here (Fig. 1).

The mechanistic driver of the discrepancy between the relevance of Fc-dependent effector functions to L9-LS- and 317-LS-conferred protection is uncertain. In contrast to the divergent results we observed with CSP mAbs, a study of protection from influenza by therapeutic mAbs reported similar loss of protection in FcgR-binding-ablated GRLR variants of three distinct mAbs that bind either influenza HA or NA protein (18). Notably, the viral antigenic targets of therapeutic antibodies for influenza and HIV are surface proteins expressed at relatively sparse levels on viral particles. In contrast, CSP is a highly abundant surface protein offering a plethora of mAb-binding sites. This phenomenon is further amplified by the preferential binding of the mAbs studied here on repeat motifs within CSP. When engaged with antigen, CSP repeat-binding mAbs extensively interact with other engaged mAbs through homotypic interactions and can also influence both proteolytic cleavage and conformational folding of CSP (35, 43–46). FcgR clustering is functionally important for signaling and effector function (47). We posit that FcgR cluster engagement may be differentially impacted by the complex geometries and stoichiometries produced by mutually interacting mAbs bound to densely packed CSP motifs, thereby leading to differential effector function outcomes for various CSP mAbs.

To circumvent limitations imposed by the limited number of parasites that can be harvested from infected mosquitoes, our *in vitro* characterization of mAb effector function activity largely relied upon assays that used beads or plates coated with rCSP. To understand the limitations of this strategy, we exploited previously described differences in the folding and cleavage of CSP in immature parasites harvested from mosquito midguts, vs mature parasites harvested from mosquito salivary glands (37), to compare the functional activity against different forms of CSP. We observed that the mAbs L9-LS and CIS43-LS more densely bound immature midgut parasites. This differential binding was not observed with the mAb 317-LS (Fig. 2D and E). One possible explanation for the similar binding of L9 and CIS43, but not 317, is their preferred binding regions: L9 and CIS43 preferentially bind at or near the N-terminal junction leading into the NANP repeat region (9, 35, 36), while 317 preferentially binds the major NANP repeats (45). Importantly, our assay measuring neutrophil phagocytosis of live parasites showed that mAbs more effectively enhanced phagocytosis of midgut parasites, compared to salivary gland parasites (Fig. 3I), supporting the functional relevance of the observed differences in mAb binding to live parasites. Our live parasite neutrophil phagocytosis assay also demonstrated significant enhancement of phagocytosis upon introduction of GAALIE and AFUC modifications to the CIS43-LS Fc (Fig. 3I), likely via increasing binding to FcgR3a. The same modifications enhanced phagocytosis in our rCSP-coated bead assay (Fig. 3C through G). In total, our data suggest that rCSP-based approaches can be used as a high-throughput strategy to discern physiologically relevant differences in the effector function activity of CSP mAbs. However, subtle differences in CSP conformations are also likely to influence CSP mAb binding with implications for immune effector function and are thus worthy of further consideration. Some of our findings were confirmed by Kisalu et al., showing that Fc engineering to increase FcgR3a binding had the potential to augment phagocytosis of CSP-coated beads via neutrophils or monocytes for CIS43-LS, but not L9-LS (42). This publication, however, did not examine the more relevant phagocytosis of live parasites. Notably, our dual assays measuring neutrophil phagocytosis of rCSP-coated beads (Fig. 3E through H) and live parasites (Fig. 3I) revealed that CSP mAbs more potently drove phagocytosis of live parasites, vs rCSP-coated beads. Along the same lines, we show that NK cell activation (IFNg and CD107a expression), via CSP-coated plates or the killing of CSP-pulsed target cells, was abrogated for all three mAbs (317-LS, CIS43-LS, and L9-LS) when the binding of the Fc to FcgRs was abrogated (N297A mutation), but was enhanced when binding to FcgR3a was increased (AFUC or GAALIE mutation). Some of these data were confirmed by Kisalu et al., in a less comprehensive analysis (42). Accordingly, we suggest that scalable assays relying on rCSP reagents may understate the potency of CSP mAbs in driving effector function. Under *in vivo* conditions where therapeutic mAb concentrations will wane over time, it is possible that mAb-driven effector function could extend the protective in-life period by conferring protection after mAbs have waned to sub-neutralizing levels.

The work presented here provides two major pieces of evidence to support the promise of pursuing the strategy of enhancing CSP mAb therapeutic efficacy via Fc engineering. First, modification of the Fc region of multiple CSP mAbs modulated mAb-FcgR binding, with expected functional consequences in driving immune effector functions. Second, Fc-dependent effector function can meaningfully contribute to *in vivo* protection of a CSP antibody. Our detection of Fc-dependence of protection for the 317-LS mAb, but not L9-LS, combined with our observations of differential potency of 317-LS, L9-LS, and CIS43-LS in driving effector function *in vitro*, calls for further mechanistic studies of Fc-dependent effector protection driven by CSP mAbs to support the rationale design of Fc-engineered CSP therapeutic antibodies. Importantly, our *in vivo* studies only discerned dependence of 317-LS protection when challenge was performed via mosquito bite, and not after intravenous inoculation of parasites (Fig. 1A through C). The restriction of Fc-dependence of 317-LS protection to the mosquito bite model suggests that antibody-driven effector function suppresses infection by targeting parasites as they transit through the skin on their journey to the liver. This model is consistent with reports that CSP mAbs exhibit superior protection against mosquito bite challenge, compared to intravenous challenge (26). Accordingly, we are eager to see future work investigate the effector function activity against parasites in the skin dermis.

Strong protection of children via prophylactic administration of L9-LS mAb in a recent Ph2 trial (2) places L9-LS as a highly intriguing candidate for Fc-engineering. Our *in vivo* studies were able to detect a contribution of Fc-dependent effector function to protection conferred by 317-LS, but not L9-LS. However, it remains uncertain if Fc-enhanced variants of L9-LS could further improve upon the potent protection of L9-LS. Our *in vitro* studies showed that Fc-engineering can enhance L9-LS to drive diverse effector functions. Hence, Fc-engineering of the already potent L9-LS mAb is an exciting avenue for further investigation.

In contrast, the 317-LS mAb has not yet been tested in humans. However, a CSP binding antibody, MAM01, that shares the NANP repeat motif binding preference of 317-LS, provides protection in mouse models, and is further engineered with enhanced developability characteristics, was recently described (8). If the NANP repeat-binding motif of 317-LS drives the functional differences we observed between 317-LS and junctional binders L9-LS and CIS43-LS, we suggest that the MAM01 mAb is also a highly appealing candidate for Fc-engineering. Our studies provide evidence that Fc-dependent effector functions contribute to the protection of an NANP repeat-binding mAb. In addition, Fc-engineering modulated the binding and *in vitro* effector function activity of NANP repeat-binding 317-LS mAb. Notably, the baseline potency of 317-LS in driving *in vitro* effector function was relatively low compared to that of L9-LS and CIS43-LS, suggesting the potential that Fc-engineering strategies may have more room to improve the activity of NANP repeat-binding 317-LS mAb. Finally, 317-LS was the only CSP mAb for which we detected mAb-driven enhancement of T-cell activation following CSP immune complex uptake and presentation by DCs (Fig. 6). Antibody enhancement of T-cell responses remains poorly understood mechanistically. However, a study focused on influenza therapeutic antibodies similarly showed that Fc-engineering with a GAALIE modification-enhanced T-cell-driven adaptive responses by modulating the DC activity (18). A therapeutic malaria mAb that could bridge innate and adaptive immunity and thereby offer long-lasting vaccine-like protection would greatly bolster the appeal of pursuing therapeutic antibodies as a malaria control strategy.

The work presented here provides evidence that CSP mAbs can be engineered for enhanced engagement of immune effector functions via modification of the Fc region. Follow-up studies that explore the mechanisms by which Fc-engineered CSP mAbs engage in innate and adaptive immunity and thereby increase potency have the potential to support the development of anti-malarial therapeutic antibodies with enhanced efficacy and practicality for deployment in resource-limited malaria-endemic regions.

## MATERIALS AND METHODS

### Antibody production

The amino acid sequences of the variable region of the anti-malaria CSP antibodies CIS43 (WO2018148660A1), L9 (WO2020227228A2), and 317 (340195023 A1) were retrieved from patients, and the reverse-transcribed nucleotide sequences were synthesized by Genscript using codon optimization for expression in hamster cells. The synthetic variable regions were subcloned into the proprietary expression vector system (pVIR-G1m3/Kappa) in frame with the appropriate heavy- and light-chain constant regions; for the heavy chain, either IgG1m3, IgG1m3-LS (M428L and N434S), IgG1m3-LS-GA (M428L, N434S, and G236A), IgG1m3-LS-N297G (M428L, N434S, and N297G), IgG1m3-LS-GAALIE (M428L, N434S, G236A, A330L, and I332E), or IgG1m3-LS-EFT (M428L, N434S, S367E, H268F, and S324T) were used. Kappa allotype Km3 (alanine 153, valine 191) was used for the light chain of all antibodies. The heavy- and light-chain vectors were co-transfected in ExpiCHO-S cells (ThermoFisher, A29133) according to the manufacturer’s protocol.

Afucosylated antibodies were produced by supplementation of 100 uM SGN-2FF (2-fluorofucose peracetate, MedChem Express, HY-107366-100MG) in the cell culture starting 1 day prior to transfection. Cell culture supernatants were harvested at day 8 post-transfection and clarified by centrifugation at 4,000 rpm for 30 min, followed by 0.22 um filtration. The recombinant antibodies were purified from filtered cell culture supernatants using protein A affinity chromatography (HiTrap MabSelect PrismA, GE Healthcare), followed by desalting (HiPrep 26/10 Desalting) equilibrated in 20 mM histidine, 50 mM NaCl, 8% sucrose, pH 6.0. Chromatography was performed using an Äkta Pure FPLC system operated with Unicorn 7.8 (Cytiva).

Antibody concentrations were determined based on protein A affinity using Biosensor Protein A pins (Pall, ForteBio) on Octet HTX (Sartorius) or Nanodrop.

### Mice

Female humanized FcgR mice generated on a C57BL6/j background were obtained from genOway. In the genOway hFcgR mice, the mouse FcgR alleles were replaced with human variants, including hFcgR1, hFcgR2b (I332), hFcgR2a (R131), hFcgR3a (F158), and hFcgR3b, with a CRISPR engineering strategy. Mice were 7–14 weeks old at experimental onset. During experiments, mice were maintained at an in-house vivarium at Vir Biotechnology. All experimental protocols were approved by the Washington University School of Medicine Institutional Animal Care and Use Committee.

### Isolation of sporozoites

Mosquitoes infected with *P. berghei* Pb-*Pf*CSP-*Luc* parasites (23) were produced by the Johns Hopkins Malaria Research Institute Parasitology Core and shipped to a Vir facility.

To isolate parasites, infected mosquitoes were manually dissected, using forceps to disrupt insects between the thorax and salivary gland-bearing heads. Materials were collected into DMEM supplemented with penicillin-streptomycin and 0.1% BSA on ice. Parasites were differentially purified for *in vitro* and *in vivo* experiments, as detailed below. For all experiments, purified viable parasites were counted on a hematocytometer following trypan blue staining.

For *in vitro* experiments, mature salivary gland parasites were harvested from salivary glands, and immature midgut sporozoites were isolated from the thorax. Materials were separately collected on ice and then serially disrupted by manual homogenization through a series of cell strainers (100 micrometer followed by 40 micrometer). The parasite-bearing material was pelleted via centrifugation at 400 *g* and then isolated on a 20% Accudenz gradient as previously described (48).

For *in vivo* experiments, mature salivary gland parasites were harvested from salivary glands by spinning through glass wool columns, as previously described (49).

### Mouse challenges

For intravenous challenge, 2 × 10^3^ sporozoites isolated from mosquito salivary glands were injected at the retroorbital cavity.

For mosquito bite challenges, female mosquitoes were aliquoted into mesh-covered conical tubes 1 day prior to challenge and starved overnight (18–22 hours). Prior to the challenge, each tube was confirmed to contain 6–8 viable female mosquitoes. Mice were anesthetized with avertin while maintaining temperature with heat packs and exposed to mosquito bites on shaved abdomens for 15 minutes. Every 5 minutes, tubes were rotated between mice so that each mouse was exposed to bites of mosquitoes from three different tubes throughout the challenge. Mosquitoes were visually confirmed to be actively feeding on mice and show evidence of a blood meal post-challenge. After the mouse challenge, mosquito salivary glands were dissected and confirmed to contain sporozoites, typically 1.000–4.000 sporozoites/mosquito. A subset of experiments where mosquitoes harbored very few salivary gland sporozoites (<800/mosquito) or failed to robustly feed on mice during the challenge were excluded from our analyses.

Prophylactic mAb treatments were given 1 day prior to mosquito bite challenge, via intravenous injection in the tail vein. Serum samples were collected 5–6 days post-challenge to confirm drug on board.

### Parasite burden by IVIS

Parasite burden was determined via IVIS imaging on an IVIS SpectrumCT of isoflurane-anesthetized mice 15 minutes after intraperitoneal (IP) injection of VivoGlo luciferin (ProMega). Mice were imaged 40–44 hours post-challenge to assess liver-stage parasite burden and 5 days post-challenge to assess early blood-stage parasite burden. Quantitative analysis of bioluminescence was performed by measuring the luminescence signal intensity using the ROI settings of the *Living Image* software. ROIs were placed around the liver or the whole mouse, as described in the figure legend. Values were normalized to the value of the mAb-untreated IV challenge control group from each experiment. Data were analyzed via one-way ANOVA with Kruskal-Wallis post-test.

### Quantification of mAbs in mouse serum

Antibody on board for mouse challenges was confirmed by quantifying antibody content in mouse serum at day 5 post-challenge (day 6 post-antibody treatment). Serum samples were analyzed with an MSD-based electrochemiluminescence assay, using the AbD34205-my-IgG2a as a capture antibody specific to the LS of each antibody and ruthenium-tagged mouse anti-human IgG CH2 domain as the detection antibody. The concentration of antibody in each sample was inferred via fitting to a standard calibrator curve with a 4PL model and weighting factor of 1/Y^2^.

### Opsonization and viability of sporozoites *in vitro*

Sporozoites isolated on an Accudenz gradient were resuspended in RPMI supplemented with 0.5% BSA. Then, 1 × 10^4^ sporozoites per well were transferred into 96-well V-bottom plates on ice. After the addition of antibodies to sporozoite-containing wells, plates were centrifuged at 2,500 *g* for 7 minutes and then incubated for 15 minutes at 37°C with shaking at 280 rpm. Plates were then spun again for 5 minutes at 2,500 *g,* and supernatants were discarded. The LIVE/DEAD Fixable Far Red Dead Cell Stain Kit was used to stain sporozoites with a 30 minute incubation on ice. Plates were then washed with PBS, followed by fixation with 2% formaldehyde for 30 minutes on ice. Plates were finally incubated with anti-human IgG1-Brilliant Violet 786 secondary antibody (BD Biosciences) to detect CSP antibodies bound to parasites. Stained samples were washed with PBS and analyzed on a Cytoflex flow cytometer.

### Phagocytosis of sporozoites *in vitro*

Sporozoites isolated on an Accudenz gradient were resuspended in RPMI supplemented with 0.5% BSA and transferred to V-bottom 96-well plates, using 1 × 10^4^ sporozoites per well. After the addition of antibodies to sporozoite-containing wells, plates were centrifuged at 2,500 *g* for 7 minutes and then incubated for 25 minutes at 37°C with shaking at 280 rpm. A total of 10,000 neutrophils freshly isolated from human peripheral blood were added to sporozoite-mAb samples. Samples were centrifuged at 2,500 *g* for 5 minutes and then co-incubated for 25 minutes at 37°C. After the phagocytosis co-incubation, plates were again centrifuged at 2,500 *g* for 5 minutes and stained with the LIVE/DEAD Fixable Far Red Dead Cell Stain Kit (Invitrogen) for 30 minutes on ice. Stained samples were centrifuged at 2,500 *g* for 5 minutes, and supernatants were discarded, followed by fixation with 2% paraformaldehyde for 30 minutes on ice. Samples were washed with PBS and analyzed on a Cytoflex flow cytometer.

Neutrophils were harvested from freshly drawn blood harvested through an IRB-approved study protocol at the Saint Louis University Center for Vaccine Development (IRB # 32287).

### Binding analysis of CSP mAbs to receptors

Lyophilized biotinylated FcgR proteins were purchased from Sino Biological and reconstituted according to the manufacturer’s protocol. Reconstituted FcgR proteins were then diluted in the running buffer 1 x HBS-EP +pH 7.4 (10 mM HEPES, 150 mM NaCl, 3 mM EDTA, and 0.05% vol/vol Surfactant P20) (Cytiva, Cat #: BR100669) to different final concentrations (See Table 1).

**TABLE 1:** Preparation of FcgR proteins used in this study

Protein name^*a*^	Catalog number^*b*^	Contact time (s)^*c*^	Final conc. (ug/mL)^*d*^
FcgR2a(H) F	10374-H27H1-B	12	0.5
FcgR2a(R)	10374-H27H-B	12	0.5
FcgR2b	10259-H27H-B	60	0.5
FcgR3a(V)	10389-H27H1-B	60	1.5
FcgR3a(F)	10389-H27H-B	25	0.5
FcgR3b Biotinylated	11046-H27H-B	180	2

^*a*^ Lyophilized protein description.

^*b*^ Purchased from Sino Biological.

^*c*^ Time in seconds for reconstitution of the lyophilized material.

^*d*^ Concentration in running buffer (10 mM HEPES, 150 mM NaCl, 3 mM EDTA and 0.05% vol/vol Surfactant P20).

Measurements were performed using a Biacore T200 instrument. Series S Sensor Chip CAP (Cytiva, Cat #: 28920234) was used to capture the biotinylated FcgR proteins. To optimize for capture levels, FcgR proteins were diluted to different concentrations and captured for different amounts of time (See Table 1). Experiments were performed with a 5-point fourfold dilution series of CIS43 IgGs with wild-type or Fc modifications.

Experiments were run as single-cycle kinetics with *n* = 2 replicates for each FcgR-IgG pair. Data were double reference-subtracted and fit to a binding model using the Biacore Insight software. The 1:1 binding model was used to determine the kinetic parameters.

### Binding of CIS43 to C1q complement component by BLI

CIS43-rIgGs bearing a wild-type or modified Fc were diluted at 10 ug/mL in kinetics buffer (PBS 0.01% BSA pH 7.1) and captured on pre-hydrated anti-human Fab CH1 (Sartorius, Cat #18-5125) for 10 min. Sensors were moved into wells containing C1q (Sigma-Aldrich Cat #C1740-5 MG), prepared in kinetics buffer at 3 ug/mL, and association/dissociation steps recorded for 4 min each using an Octet-HTX (Sartorius) instrument. The 1:1 binding model was used to determine the kinetic parameters.

### Transgenic T-cell reporter assay for stimulation by immune complexes

To assess antigen-specific T-cell responses to immune complexes, a triple reporter Jurkat line (41) was engineered to overexpress a human TCR specific for CSP (40). Antigen-specific stimulation was performed using HLA-matched (DRB1*07:01P) monocyte-derived dendritic cells (moDCs) obtained through differentiation of primary CD14 monocytes isolated from healthy PBMCs (StemCell) and treated with 100 ng/mL IL4 and 100 ng/mL GM-CSF (PeproTech) for 6 days. Immune complexes were generated by mixing 100 ug/mL mAbs with 6 ug/mL recombinant CSP for 30 minutes at room temperature prior to addition to antigen-presenting cells. Following moDC stimulation, Jurkat CSP TCR cell lines were added at a 1:1 ratio overnight, and NFAT-GFP reporter expression and CD69 upregulation were analyzed by flow cytometry.

### Coating of beads with rCSP for assessment of effector function assays

For bead-based assays (antibody-dependent phagocytosis and antibody-dependent complement deposition), a two-step carbodiimide reaction was used to couple recombinant CSP to either yellow-green or blue carboxylate-modified beads (Invitrogen, F8823/F8815). Beads (10 µL) were activated for 30 minutes at RT using 100 mM monobasic sodium phosphate, pH 6.2 with 50 mg/mL sulfo-NHS (N-hydroxysulfosuccinimide, Pierce, A39269) and 50 mg/mL ethyl dimethylaminopropyl carbodiimide hydrochloride (EDC). Beads were then washed with 50 mM MES, pH 5.0, and incubated with 20 µg of antigen in 50 mM MES, pH 5.0, for 2 hours on a rotator. Finally, the coupled beads were blocked with 5% BSA in PBS overnight at 4°C and then washed and resuspended in 0.1% BSA. Recombinant His-tagged CSP was produced in *Lactococcus* (B2S Life Sciences, rCSP 4/38-His Ferm3-5L) (50).

### Antibody-dependent phagocytosis of rCSP-coated beads by THP-1 monocytes

Yellow-green rCSP-coupled beads (10 µL/well) were added to 96-well plates with 10 µL/well of diluted mAb sample (diluted in PBS) and incubated for 2 hours at 37°C to form immune complexes. After incubation, the immune complexes were washed with PBS, and THP-1 cells (2.5 × 104 cells/well) were added and incubated for 16 hours at 37°C. After incubation, the supernatant was removed, and cells were fixed with 4% PFA for 15 minutes. Fluorescence was acquired with a Stratedigm S1000EON. The mAbs were tested at a range of 5 µg/mL to 2.28624 ng/mL.

### Antibody-dependent phagocytosis of rCSP-coated beads by neutrophils

Yellow-green rCSP-coupled beads (10 µL/well) were added to 96-well plates with 10 µL/well of diluted mAb sample (diluted in PBS) and incubated for 2 hours at 37°C to form immune complexes. After incubation, the immune complexes were washed with PBS, and the supernatant was removed. Neutrophils were isolated from commercially purchased freshly unemployed whole blood using the EasySep Direct Neutrophil Isolation kit (StemCell, 19666), resuspended in R10, and added to plates at a concentration of 2.5 × 104 cells/well. The plates were incubated for 30 minutes at 37°C. The cells were stained for the neutrophil marker CD66b (Biolegend, 305112), washed with PBS, and fixed for 15 minutes in 4% PFA. Fluorescence was acquired with a Stratedigm S1000EON. The mAbs were tested at a range of 30 µg/mL to 13.72 ng/mL.

### Antibody-dependent complement deposition on rCSP-coated beads

Immune complexes were formed by incubating 10 µL of blue rCSP-coupled beads with 10 µL diluted mAb sample (diluted in PBS) for 2 hours at 37°C. After incubation, the immune complexes were washed with RPMI (HyClone, SH3009601). Lyophilized guinea pig complement (Sigma, G9774) was resuspended in 5 mL of cold water, diluted 1:60 in gelatin veronal buffer (Sigma, G6514), and added to the immune complexes (150 µL per well). The plates were incubated for 50 minutes at 37°C, and the reaction was stopped by washing the plates twice with 15 mM EDTA in PBS. To detect complement deposition, plates were incubated with fluorescein-conjugated goat anti-guinea pig complement C3 (MP Biomedicals, 0855385) for 20 minutes in the dark. Fluorescence was acquired with Stratedigm S1000EON, and the data are reported as the average (*n* = 2 technical replicates) median fluorescent intensity of the FITC channel. The mAbs were tested at a range of 67 µg/mL to 30.64 ng/mL.

### Antibody-dependent activation of NK cells

Antibody-dependent NK cell activation was measured with rCSP protein-coated plates. ELISA plates were coated with recombinant CSP (300 ng/well) and incubated for 2 hours at RT. Plates were blocked with 5% BSA in PBS overnight at 4°C. Human NK cells were isolated from commercially purchased leukopaks (StemCell Technologies) using the EasySep NK cell enrichment kit. The sample was diluted in PBS, and 50 µL of the diluted sample was added to the plates. Plates were incubated for 2 hours at 37°C to form immune complexes. After the incubation, the plates were washed with PBS, and NK cells (5 × 10^4^ cells/well) in R10 supplemented with anti-CD107a PE-Cy5 (BD Biosciences, 55802), GolgiPlug (BD Biosciences, 555029) and GolgiStop (Biolegend, 420701). Plates were incubated for 5 hours at 37°C. Following the incubation, NK cells were stained with anti-CD56 PE-Cy7, anti-CD16 APC-Cy7, and anti-CD3 Pacific Blue (BD Biosciences, 557747, 557758, and 558124) and then fixed and permeabilized with the Fix&Perm cell permeabilization kit (Life Technologies, GAS001S100/GAS002S100). The cells were then stained with anti-MIP-1β PE and anti-IFNγ FITC (BD Biosciences, 550078 and 340449) to stain for intracellular markers. Fluorescence was acquired on a Stratedigm S1000EON. Each sample is tested with at least two different NK cell donors, with all samples tested with each donor. The data are reported as the individual (*n* = 2 donors) percent of cells positive for each of the activation markers (CD107a, IFN-γ, and MIP-1β). The mAbs were tested at a range of 20 µg/mL to 9.1449 ng/mL.

### Antibody-dependent cellular cytotoxicity by NK cells

Target cells were biotinylated (EZ-Link Sulfo-NHS-LC-Biotin) and stained with one of two dyes: half with CellTrace Violet and half with CellTrace Far Red (ThermoFisher, C34564, C34557). Recombinant CSP was streptavidinated using the Abcam Lightning-Link Streptavidin Conjugation kit (Abcam, ab102921). The stained cells are then pulsed with either streptavidin-conjugated recombinant CSP or left unpulsed. Diluted antibody (diluted in R10) is added in a 1:1 mixture to the target cells. NK cells purified from healthy blood donor leukopaks using the StemCell EasySep Human NK Cell Isolation Kit (StemCell, 17955) were added at a 10:1 effector:target and incubated for 4 hours. Following this incubation, the cells were stained with a viability dye (BioLegend, 423112). Fluorescence was acquired with Stratedigm S1000EON, and ADCC activity is reported as the percent specific lysis calculated using the formula: percent specific lysis = [ (% dead of pulsed cells) – (% dead of unpulsed cells)] / (100 – (% dead unpulsed cells))

The data are reported as the average (*n* = 2 donors) percent lysis, and the mAbs were tested at a range of 1 µg/mL to 0.46 ng/mL.

### Statistical analysis

Statistical analyses were performed in GraphPad Prism. Data distribution as either normal or non-normal was used to select appropriate statistical tests. All reported *P*-values were two-tailed and adjusted for multiple comparisons.
